# A Novel Lactic Acid Bacteria Mixture: Macrophage-Targeted Prophylactic Intervention in Colorectal Cancer Management

**DOI:** 10.3390/microorganisms8030387

**Published:** 2020-03-11

**Authors:** Petra Hradicka, Jane Beal, Monika Kassayova, Andrew Foey, Vlasta Demeckova

**Affiliations:** 1 Institute of Biology and Ecology, Faculty of Science, Pavol Jozef Safarik University in Kosice, Srobarova 2, 041 54 Kosice, Slovak; petra.hradicka@upjs.sk (P.H.); monika.kassayova@upjs.sk (M.K.); 2 School of Biological and Marine Sciences, University of Plymouth, Drake Circus, Plymouth PL4 8AA, UK; j.d.beal@open.ac.uk; 3 School of Biomedical Sciences, University of Plymouth, Drake Circus, Plymouth PL4 8AA, UK; andrew.foey@plymouth.ac.uk

**Keywords:** *Lactobacillus salivarius*, *Lactobacillus plantarum*, cytokines, immunomodulation, Wistar rat, 1,2-dimethylhydrazine

## Abstract

Colorectal cancer (CRC) is one of the most common forms of cancer. Its onset from chronic inflammation is widely accepted. Moreover, dysbiosis plays an undeniable role, thus the use of probiotics in CRC has been suggested. They exhibit both anti- and pro-inflammatory properties and restore balance in the microbiota. The aim of this study was to investigate the immunomodulatory properties of six lactobacilli with probiotic features in an in vitro model of macrophage-like cells and to test these pooled probiotics for their anti-tumour properties in a chemically induced CRC model using Wistar male rats. Upon co-culture of M1- and M2-like macrophages with lactobacilli, cytokine release (TNF-α, IL-1β, IL-18, IL-23) and phagocytic activity using fluorescent-labelled bacteria were tested. The effects of orally administered probiotics on basic cancer and immune parameters and cytokine concentration (TNF-α, IL-1β, IL-18) in colon tumours were studied. Tested lactobacilli exhibited both pro- and anti-inflammatory properties in in vitro conditions. In vivo study showed that the administration of probiotics was able to decrease multiplicity, volume and total tumour numbers, restore colon length (*p* < 0.05) and increase IL-18 production (*p* < 0.05) in tumour tissue. These data indicate both an immunomodulatory effect of probiotics on distinct macrophage subsets and a protective effect against chemically-induced CRC.

## 1. Introduction

Due to its metabolic activity, the human microbiota is defined as a virtual organ that consists of more than 10^13^ cells, which are found lining the skin and mucosal surfaces such as the gastrointestinal tract. The relative bacteria:human cell ratio in the literature varies from 1:1 to 10:1, nevertheless, the biological importance of the microbiota is undeniable [1,2,3]. The microbiota has also been shown to have protective and structural effects on the epithelium and is responsible for maturation of the gut mucosal immune system [4]. On the other hand, alterations in microbial composition are associated with many diseases, such as inflammatory bowel disease [5] and colorectal cancer (CRC) [6]. Dysbiosis is defined as any change in commensal communities leading to an imbalance between harmless and harmful bacterial species; such imbalances may account for either the cause or consequence of disease [7,8]. There is, however, a strong correlation between dysbiosis and gut inflammation, as evidenced by a decreased proportion of Firmicutes and increased numbers of *Bacteroides fragilis*, *B. vulgatus* and bacteria from the *Fusobacterium* genus [9,10]. This dysbiosis can be redressed by probiotics, which in effect change the microbiota within the gastrointestinal tract into more favourable species [11]. Among probiotics, lactic acid bacteria (LAB) are the most frequently used [12]. Apart from their ability to contribute to the inhibition of pathogens [13], they have been shown to have immunomodulatory effects [14,15]. 

Probiotic strains exhibit potential as dietary supplements against neoplastic transformation, through extensive effects on the host immune system [16]. Such an investigative approach is urgently required to identify immunomodulatory probiotic strains in the treatment of colon cancer, which in effect support anti-tumour responses in both innate and adaptive immune cells. Innate immune cells are highly represented in the complex ecosystem of a tumour, with macrophages being the most abundant [17]. In addition, epithelial-associated macrophages of the gastrointestinal tract, represent the largest population of these phagocytes in the body [18]. Generally, macrophages exist in two distinct stages of polarisation, M1, which exhibit a pro-inflammatory phenotype, and M2, which have an anti-inflammatory phenotype. M1s are characterised by high production of pro-inflammatory cytokines and their induction of T_h_1 immune responses whereas M2s produce anti-inflammatory mediators and drive wound healing and induce T_h_2 immune responses [19,20]. The M2 subset has also been linked to tumour-associated macrophages (TAMs), especially because of their pro-tumour angiogenic properties [21]. This polarisation of macrophage response between pro-inflammatory M1-like and anti-inflammatory M2-like is suggested to be plastic, the manipulation of which represents a targeted therapeutic regimen for controlling immunity to pathogens and neoplastic transformation and cancer. The best example of this plasticity phenomenon is a growing body of evidence that suggests that the ability of tumours to switch between macrophage phenotypes has a beneficial effect on their own proliferation over different stages of cancer development [22,23]. Probiotics have been shown to have both anti- and pro-inflammatory effects on macrophages [24,25], thus it is possible that they can manipulate the plastic switching between these phenotypes in a favourable way to tumour surveillance and protection. The role of macrophages in tumour development is complex with dynamic macrophage phenotypic plasticity determining tumour initiation, progression and metastasis. Generally, TAMs have a dual effect on tumour growth and progression, acting in either a pro- or anti-tumour manner [26]. In early stage tumorigenesis, M1 macrophages may stimulate production of pro-inflammatory mediators and cytokines, which can favour tumour cell growth. On the other hand, M2 macrophages play a pro-tumorigenic role mainly in late stage of tumours or in hypoxic areas, where they can promote angiogenesis, inhibit anti-tumour responses and also influence tumour relapse after conventional anticancer therapies [27]. This M1/M2 classification however, is an over-simplification of a functional spectrum acquired by macrophages in response to stimuli of the tumour microenvironment. Given these key roles in homeostasis and cancer, manipulation of the functional plasticity of colonic macrophages and their cytokine production represent an exciting therapeutic intervention. Many macrophage-focused anticancer approaches have been investigated, including the blockade of tumour-promotion and activation of anti-tumour effector functions [28]. Macrophage reprogramming towards an M2 anti-inflammatory subset in chronic inflammation (early onset CRC) and an M1 tumoricidal subset post-onset CRC, represents an attractive therapeutic strategy against cancer.

LAB *Lactobacillus plantarum* VD23, C28 and MS18 and *Lactobacillus salivarius* MS3, MS6 and MS16 were used in the present study. These strains have been tested previously for their probiotic features (viability under conditions of the human gastrointestinal tract, aggregation with pathogens and lactic acid production) and showed a positive effect on animal health [29,30]. These strains were patented as fermented foodstuff product [31]. Thus, the aim of this study was to investigate the in vitro immunomodulatory potential of these strains via their ability to modulate macrophage subset-specific phagocytic potential and cytokine production profiles. CRC exhibits a specific pathological sequence progression from pro-inflammatory (M1-driven) to immunosuppressive (M2-driven) stages, thus the translatability of these probiotic strains was studied using a bacterial mixture, based on the fact that some probiotic bacteria exhibit synergistic or suppressive effects on each other [32], to assess modulation of anti-tumour responses in an in vivo model of CRC. For the further use of potential probiotic products containing multiple strains, their benefits to the host must be confirmed for the specified combination and not extrapolated from the evidence for the separate probiotic strains.

## 2. Materials and Methods 

### 2.1. Macrophage Culture

The human monocytic cell line, THP-1, was obtained from ECACC and used for this study between passages 7 and 35. THP-1 cells were cultured in R10 medium composed of RPMI-1640 medium supplemented with 10% (*v*/*v*) fetal bovine serum (Gibco, Massachusetts, Grand Island, NY, USA) and 2 mM L-glutamine, in the absence of exogenously added antibiotics. Pro-inflammatory (M1-like) macrophages and anti-inflammatory (M2-like) macrophages were generated by differentiation of these monocytes in the presence of 25 ng/mL phorbol 12-myristate 13-acetate (PMA) for three days or 10 nM 1,25-(OH)_2_-Vitamin D_3_ (Sigma-Aldrich, Poole, UK), for seven days, respectively [33]. M1-like macrophages were then washed and allowed to rest in fresh R10 medium without PMA for an additional 24 h [34]. Macrophages were plated out to a final density of 5 × 10^5^ cells/500 µL/well in R10 medium in 24 flat-bottomed well tissue culture plates.

### 2.2. Bacterial Culture

Lactobacilli tested in this study are shown in Table 1 and were kindly provided by Dr. Jane Beal from internal microbiology stocks at the University of Plymouth (UK). All bacterial species were cultured in De Man Rogosa Sharp (MRS) (Sigma-Aldrich, Poole, UK) broth at 37 °C, 5% CO2 for 18–20 h until the beginning of the stationary phase. Bacterial cells were harvested by centrifugation of 4000 g for 5 min at room temperature and resuspended in R10 medium to the desired bacterial stock density.

### 2.3. Modulatory Effect of Live Probiotic Bacteria on Cytokine Production

To investigate probiotic regulation of macrophage cytokine release, different live bacterial strains were added in culture to a final density of 1 × 10^7^ CFU/mL for 18 h (determined as the optimal concentration and time period for secretion of the cytokines TNFα, IL-1β, IL-18 and IL-23) in a humidified environment at 37 °C, 5% CO_2_. Supernatants were harvested and stored at −20 °C until assayed. Release of the pro-inflammatory cytokines, TNF-α, IL-1β, IL-18 and IL-23, were analysed by sandwich ELISA using commercially available capture and detection antibodies for TNF-α (BD-Pharmingen, Oxford, UK), IL-1β and IL-23 (eBioScience^TM^, Paisley, UK) and an IL-18 human ELISA kit (Invitrogen^TM^, Paisley, UK). Protocols were followed according to the manufacturer’s instructions and compared to standard curves, using the recognised international standards available from NIBSC (Potter’s Bar, UK) over the range of 7 to 5000 pg/mL for TNF-α, IL-1β, IL-18 or 16 to 2000 pg/mL for IL-23, respectively. Colorimetric development was measured by an OPTIMax tunable microplate reader at 450 nm and analysed by Softmax Pro software, version 2.4.1 (Molecular Devices Corp., Sunnyvale, CA, USA). Cytokine secretion results are reported as the fold of induction (FOI) compared to the unstimulated macrophage control, to which an FOI of 1 was attributed.

### 2.4. Phagocytosis Assay of Live Probiotic Bacteria

To test macrophage subset phagocytosis of different bacterial strains, 5(6)-carboxyfluorescein diacetate N-succinimidyl ester (CFSE)-loaded bacteria were used. Briefly, bacterial strains were labelled with CFSE (Molecular Probes, Leiden, The Netherlands) at a final concentration of 1.5 mM in PBS for 30 min in a humidified environment at 37 °C, 5% CO2 in the dark. Concurrently, macrophages were treated with an inhibitor of actin polymerisation, cytochalasin D (Sigma Aldrich, Dorset, UK), at a concentration of 1 µg/mL for 35 min at 37 °C, 5% CO2. After incubation, bacteria were washed extensively to remove unbound CFSE and were added to a final density of 1 × 10^7^ CFU/mL (determined as the optimal concentration for phagocytosis assay) to both untreated and cytochalasin D-treated macrophages and co-cultured in a humidified environment at 37 °C, 5% CO_2_. Phagocytosis was performed for a period of six hours with four time points—one, two, four and six hours. Cells were then washed with PBS, detached with trypsin and processed through flow cytometry. A total of 5000 events were recorded on a FACSAria II flow cytometer (BD Biosciences, San Jose, CA, USA). Data were analysed with BD FACS-Diva software (BD Biosciences, San Jose, CA, USA). Degree of phagocytosis was determined as an increase in the mean fluorescence index (MFI) ratio between untreated and cytochalasin D-treated macrophages. Phagocytosis of each probiotic strain was carried out in triplicate samples for at least *n* = 3 independent replicate experiments.

### 2.5. Animals and Diet

Male Wistar rats (*n* = 56) (Velaz, Ltd., Prague, Czech Republic) were obtained at four weeks of age (mean body weight 130.45; range 114–157g), quarantined and housed in groups of four per cage at room temperature 22 ± 2 °C, 55 ± 10% humidity and a 12 h light/dark cycle. They had free access to a standard laboratory diet (Altromin 1324, Lage, Germany) and water. Food consumption for each cage was monitored daily and body weights were recorded weekly. All experiments were approved by the National Animal Ethical Committee of the Slovak Republic (license number Ro-4058/16-221) and were conducted in accordance with the European Convention for the protection of vertebrate animals used for experimental and other scientific purposes (ETS 123).

### 2.6. In Vivo Investigation of Probiotic Effects on Colorectal Cancer 

**Scheme 1:**
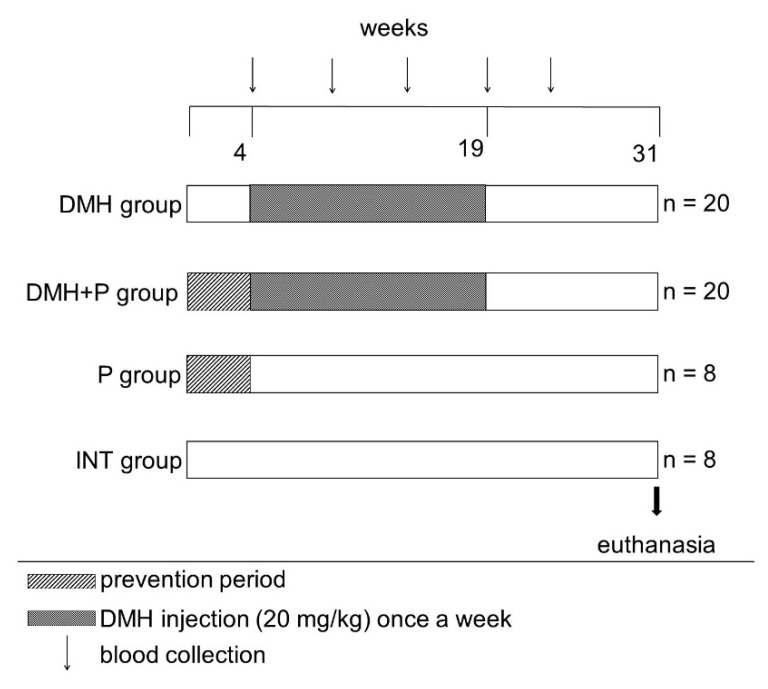
Experimental design: in vivo probiotic modulation of colorectal cancer. Four experimental groups were used in the present study (INT, control healthy group; P, probiotic-fed healthy group; DMH, control cancer group and DMH+P, probiotic-fed cancer group). Time schedule: probiotic usage as prophylactic (first four weeks, indicated by striped box – prevention period), followed by 15 weeks of DMH administration once a week (indicated by a stippled box) and a further 12-week period (from 19–31weeks), that was determined to be sufficient for tumour development. The arrows at the top indicate blood collection from the tail vein. An arrow at the bottom of the protocol indicates animal euthanasia at Week 31 at the conclusion of the experimental protocol.

After one week of acclimatisation, rats were randomly divided into four groups (Scheme 1): control cancer group (*n* = 20) (DMH), probiotic-fed cancer group (*n* = 20) (DMH+P), probiotic-fed healthy group (*n* = 8) (P) and control healthy group (*n* = 8) (INT). Probiotic-fed groups were given a mixture of LAB (LP VD23, C28 and MS18 and LS MS3, MS6 and MS16) from Monday to Friday until the end of experiment. The probiotic mixture was prepared according to standard procedure (see Section 2.2 on bacterial culture) and administered orally in a final concentration of 10^9^/50 μL/dose in sterile water once a day. The proportion of bacteria in the mixture was equal. The probiotic mixture was used as prophylactic for four weeks (prevention period) prior to 1,2-dimethylhydrazine (DMH) administration. After the prevention period, cancer groups received subcutaneous injection of DMH. CRC was induced by DMH (Fluka Chemie, Buchs, Switzerland) prepared according to the standard method [35]. DMH was dissolved in 0.9% NaCl containing 1 mM EDTA and adjusted to pH 6.8 with 1 M NaOH and administered subcutaneously at 20 mg/kg once a week for 15 weeks. A fresh solution was prepared prior to each application. Blood samples were collected monthly from the tail vein in EDTA-containing tubes (Sarstedt, Nümbrecht, Germany) until the fifth month of the study and analysed for leukocyte count on BC-2008 VET automatic analyser (Mindray, Shenzhen, China). At the age of 35 weeks, the rats were humanely euthanised by decapitation. For clarity, the experimental design is shown in Scheme 1 below.

### 2.7. Measurement of Probiotic Effects on Tumour Incidence, Colon Length and Tumour Cytokine Production

During autopsy, the colon was removed and its length was measured. It was opened along the longitudinal axis, flushed with saline and examined macroscopically for tumours, the number, size and distribution of which were recorded. Tumour size was measured by digital calipers and volume calculated by the following formula V = π∗L∗W∗H/6 (where V stands for volume, L for length, W for width and H height) [36]. Tumours were snap frozen on dry ice and stored at −80 °C until analysis. Analysis of TNF-α, IL-1β and IL-18 was performed by Bio-Plex^®^ Multiplex Immunoassay (Bioclarma Sr.l., Turin, Italy). Briefly, tissue samples were lysed according to the manufacturer’s instructions. All samples were then dosed with DC Protein Assay and diluted to a working concentration of 0.9 μg/μL. Fifty μL of this solution were analysed in each well (each sample was tested in a technical duplicate). The standard curve was optimised for lysate analysis with eight points according to manufacturer’s instructions over the range of 10.61 to 11,327.7 pg/mL for TNF-α, 3.49 to 14,002.96 pg/mL for IL-1 β and 3.56 to 9,048.72 for IL-18.

### 2.8. Statistical Analysis

All statistical analyses were performed using Minitab version 16 software (Minitab Inc., 2013, Coventry, UK). All data were examined for normal distribution, and appropriate tests applied. Statistical differences between groups were analysed using ANOVA followed by the Tukey post hoc test or Kruskal–Wallis test followed by pairwise multiple comparison procedures (Mann–Whitney test). All differences were considered statistically significant when *p* < 0.05.

## 3. Results

### 3.1. In Vitro Responses of Macrophages

#### 3.1.1. Probiotic Induction of Macrophage Cytokine Profiles was Both Strain- and Subset-Dependent

**Figure 1:**
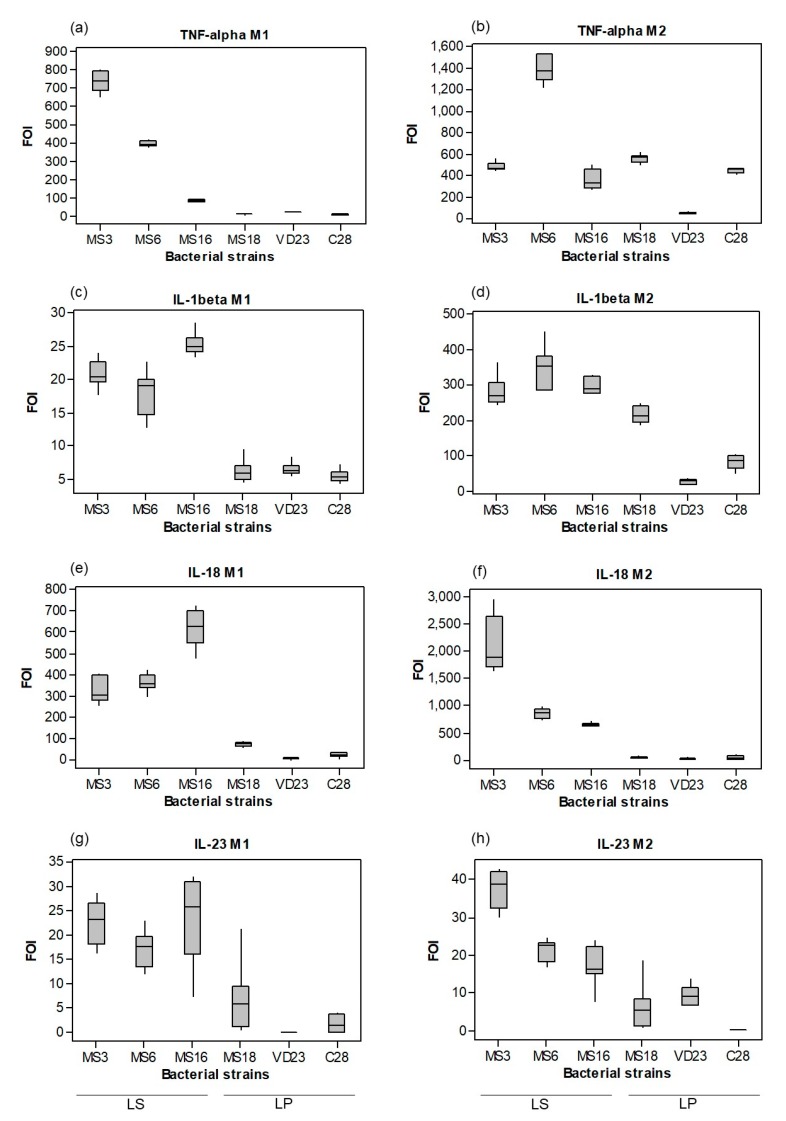
Live probiotic bacteria induce distinct proinflammatory cytokine profiles. THP-1 monocytes were differentiated into M1-like and M2-like macrophages with either 25 ng/mL PMA for three days or 10 nM 1,25-(OH)_2_ Vitamin D_3_ for seven days, respectively. M1 (**a**,**c**,**e**,**g**) and M2 (**b**,**d**,**f**,**h**) were stimulated by live probiotic strains indicated (*Lactobacillus salivarius* strains MS3, MS6 and MS16; *Lactobacillus plantarum* strains MS18, VD23 and C28), at a moi of 100:1 bacteria:macrophage cells for 24 h. Secretion of the cytokines TNF-α (**a** and **b**), IL-1β (**c** and **d**), IL-18 (**e** and **f**) and IL-23 (**g** and **h**) is expressed as the fold of induction (FOI) ratio relative to expression by the control (unstimulated macrophages), which was set at a value of 1. Box plots represent the first to the third quartile of the data. The thick line is the median, and the whiskers in the box plot extend to the minimal and maximal values. Data displayed is a representative experiment with triplicate samples of *n* = 3 replicate experiments.

Macrophage polarisation is highly dynamic. Responding to micro-environmental cues, macrophages can rapidly switch from one phenotype to the other. The abilities of six different lactobacilli strains to induce TNF-α, IL-1β, IL-18 and IL-23 cytokine release from polarised macrophages were compared (Figure 1). Lactobacilli strains differed in their ability to stimulate TNF-α production in M1 and M2 cells. In M1s (Figure 1a), three of the weakest inducers, LP VD23, MS18 and C28, differed significantly (*p* < 0.001) compared to the best inducers, LS MS3 and MS6. Although strain MS16 induced TNF-α secretion (84 FOI), it was markedly lower, compared to MS3 (740 FOI) and MS6 (400 FOI), respectively. Different results were observed for the M2 macrophage subset (Figure 1b). All strains, except for VD23, strongly stimulated TNF-α release (ranging from 360–1410 FOI), with the most potent inducer being strain MS6. VD23 induction of TNF-α secretion was low in both M1 and M2 subsets, thus was not influenced by the macrophage polarisation status. A similar trend was observed for IL-1β, especially in M2 macrophages, where cytokine release induced by LS strains MS3, MS6 and MS16 and the LP strain MS18 were the strongest within the panel of probiotic strains (Figure 1d). In M1s, similarly to M2s, the LS strains (MS16, MS3, MS6) showed the highest induction of this cytokine (Figure 1c). Again, this trend of LS strains being the highest inducers of cytokine was repeated for M1 and M2 secretion of IL-18 (Figure 1e,f), whereas MS18, VD23 and C28 induced the smallest cytokine response. Finally, the same strains that were good inducers of IL-1β and IL-18 secretion, emerged as potent inducers of IL-23, regardless of macrophage subset. LP strains MS18, VD23 and C28 were poor inducers, whereas LS strains were potent inducers of IL-23 (Figure 1g,h). 

#### 3.1.2. Phagocytic Activity of Macrophages was Strain- and Subset-Dependent 

**Figure 2:**
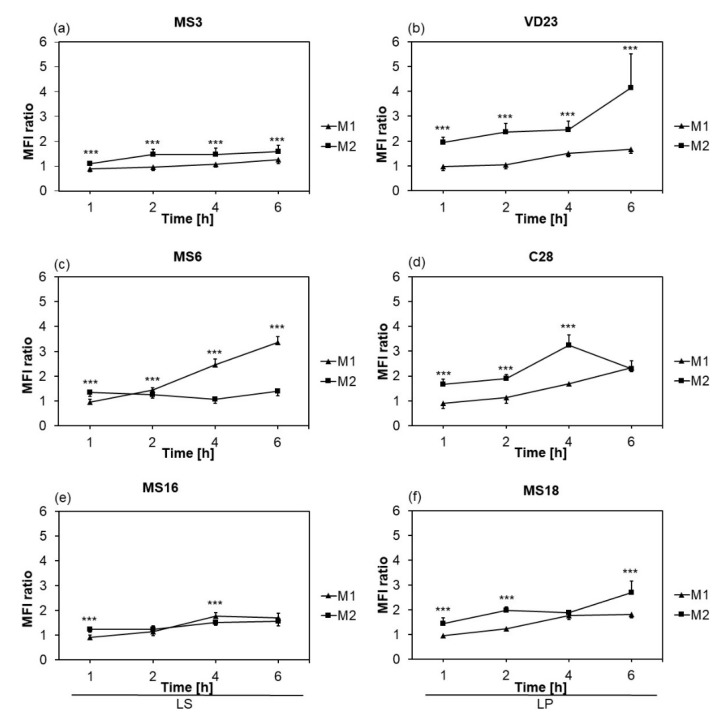
Macrophage phagocytosis of probiotic bacteria is strain-specific and subset-dependent. THP-1 monocytes were differentiated into M1-like and M2-like macrophages with either 25 ng/mL PMA for three days or 10 nM 1,25-(OH)_2_ Vitamin D_3_ for seven days, respectively. M1 (triangles) and M2 (squares) were incubated in the presence of live probiotic strains indicated, at a moi of 100:1 bacteria:macrophage cells for up to six hours in the presence or absence of 10 µg/mL cytochalasin D. Phagocytosis was measured over a six hour time-course and expressed as the mean of the median fluorescence intensity (MFI) ratio between macrophages and macrophages pre-treated with cytochalasin D ± SD at one, two, four and six hours. Diagram (**a**) shows phagocytosis of *L. salivarius* strain MS3; (**b**) *Lactobacillus plantarum* VD23; (**c**) *L. salivarius* MS6; (**d**) *L. plantarum* C28; (**e**) *L. salivarius* MS16 and (**f**) *L. plantarum* MS18. Data displayed is a representative experiment with triplicate samples of *n* = 3 replicate experiments. Significant differences in phagocytosis between M1 and M2 macrophage subsets are indicated as ** *p* < 0.01; *** *p* < 0.001.

Different phagocytic activity is another important feature of macrophage polarisation. The phagocytic index (presented as the MFI ratio) of M1- and M2-like macrophages triggered by different *Lactobacillus* strains was investigated over a six-hour time course. Generally, M2-like macrophages had significantly (*p* < 0.001) higher phagocytic properties against each strain immediately after the first hour of co-culture but with a different course over six hours (Figure 2). Based on the MFI ratio at the latest time point, the highest phagocytic activity of M2s was induced by strain VD23 (4.14 ± 1.37); M1s were significantly less phagocytic than M2s against strain VD23 at all time points (Figure 2b). Comparing the phagocytic properties of M1s, it can be seen that only strain MS6 induced significantly higher phagocytosis (*p* < 0.01) after two hours of co-culture (1.45 ± 0.8), which increased to 3.35 ± 0.25 after 6 h (Figure 2c). Interestingly, M1s displayed a higher phagocytic index against this strain than M2s. After four and six hours there was even two to three-times stronger phagocytic activity in M1s than M2s (*p* < 0.001) (Figure 2c). LS MS3 (Figure 2a) and MS16 (Figure 2e) induced very low phagocytic activity in both M1s and M2s, although M2 macrophages were slightly more phagocytic (*p* < 0.001 and *p* < 0.01, respectively). Significantly stronger phagocytic activity (*p* < 0.001) was observed after one, two and six hours of co-culture of M2s with MS18, when compared to M1s (Figure 2f). LP C28 induced significantly stronger phagocytic activity in M2s than in M1s at all time points (*p* < 0.001), where after four hours the ratio for C28-treated M2s increased to 3.24 ± 0.41, apart from six hours, where the MFI ratio in M2s dropped to the level of M1s. Phagocytic activity of C28 by M1s linearly increased over time with a maximal index reached at six hours (Figure 2d). Finally, it can be concluded that the lowest phagocytic activity in M1s was induced by strains MS3 and MS6 and for M2s by strain MS3. On the other hand, the highest phagocytic activity in M1s was induced by MS6 and in M2s by VD23.

### 3.2. In Vivo Anti-Tumour Effect of LAB Mixture

#### 3.2.1. Animal Health Status

Subcutaneous injection of DMH was well tolerated, as no clinical signs of toxicity were observed. All rats appeared healthy and gained weight throughout the experimental period (Table 2). Although the DMH+P group showed statistically significant reduction in food consumption compared to all other experimental groups (*p* < 0.001), there was no significant difference in food efficiency ratio (FER) (Table 2). Both probiotic-fed groups did not show a significant difference in FER, suggestive of an unaffected ability to convert food in body mass by DMH treatment in the DMH+P group. Moreover, colon length was significantly reduced by DMH injection and rescued upon administration of probiotics. The probiotic-fed group did not alter the length of the colon compared to control, while colon length in the DMH group was decreased in comparison with all groups tested (*p* < 0.05). Colon length of rats in the DMH+P group did not show any significant difference compared to both P and INT groups, whereas it demonstrated a significant increase over the DMH group, indicating that orally administered probiotics were able to restore colon length (Table 2). 

Macroscopic analysis of the colon showed a 50% tumour incidence in both DMH administered groups (Table 3). In the DMH group, the total number of tumours was nine, while in the DMH+P group, the number of tumours dropped to six. Moreover, average tumour volume in the DMH group was 2.42 ± 0.56 cm^3^, while in the DMH+P group it was 1.66 ± 0.64 cm^3^. Although, these are not significant differences, there is a trend in the reduction of multiplicity and volume after probiotic treatment. This trend in reduced tumour involvement was further reinforced by a reduced tumour multiplicity score and a reduction in the number of tumours observed in both the proximal and distal colon upon probiotic treatment of DMH-administered animals. 

#### 3.2.2. LAB Mixture Treatment had a Selective Effect on Tumour Tissue Secretion of Cytokines.

**Figure 3:**
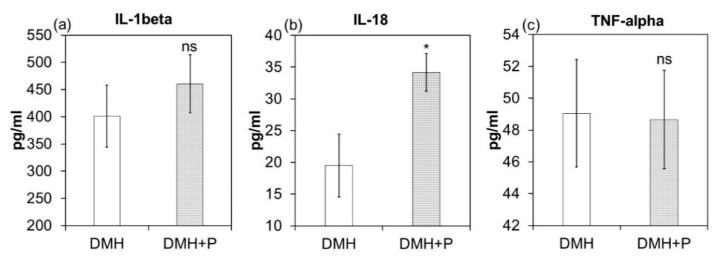
Probiotic treatment differentially modulated tumour IL-1β, IL-18 and TNF-α. Standardised tumour tissue lysate obtained from DMH, untreated colorectal cancer (unshaded) and DMH+P, probiotic treated colorectal cancer (stippled) was analysed for pro-inflammatory cytokine production by multiplex immunoassay. IL-1β (**a**), IL-18 (**b**) and TNF-α (**c**) are expressed as the mean ± SEM in pg/mL, for *n* = 6 tumour samples per group run in duplicate. Significant differences in cytokine production between DMH and DMH+P are indicated as ns, not significant and * *p* < 0.05.

IL-1β, IL-18 and TNF-α are macrophage-derived cytokines that have been proven to display pro-inflammatory activity. Mean cytokine levels in tumour tissue for both cancer groups are shown in Figure 3. LAB treatment increased IL-1β secretion in tumour tissue whereby the DMH+P group (460.2 ± 53.5 pg/mL) was higher than those in the cancer control (401.1 ± 56.9 pg/mL), but without statistical significance (Figure 3a). Probiotic treatment significantly increased IL-18 production (*p* < 0.05) (Figure 3b), whereas TNF-α levels were not significantly influenced (49.05 ± 3.39 pg/mL in the DMH group and 48.66 ± 3.1 pg/mL in the DMH+P group) (Figure 3c). 

#### 3.2.3. LAB Mixture Treatment had a Positive Effect on Leukocyte Count 

**Figure 4:**
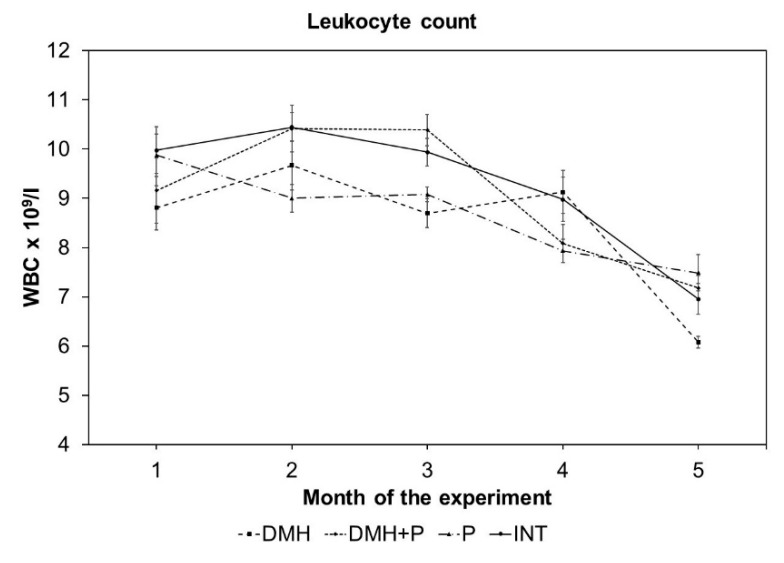
Leukocyte count decreased over the experimental period. Data are displayed as mean ± SEM, *n* = 6 rats per group. No statistical significance was observed at 1, 2, and 4 months versus DMH group, *p* < 0.001 DMH versus DMH+P, *p* < 0.01 DMH versus INT at the third month, and *p* < 0.001 DMH versus DMH+P and *p* < 0.05 DMH versus INT group at the fifth month.

Leukocyte cell count was declining with increasing age of experimental rats (Figure 4). After the prevention period, there were no significant differences in leukocyte count between experimental groups. Interestingly, the first significant decrease (*p* < 0.05) in leukocyte count was observed in the probiotics-fed (P) group at the second blood collection time point compared to the INT group. Administration of DMH induced a statistically significant decrease in leukocyte count after three months of experiment (*p* < 0.01 vs. INT, *p* < 0.001 vs. DMH+P), however, probiotic feeding during cancer progression protected animals from the leukocyte drop, not only at this blood collection time point, but during the entire experimental period. The animals in the cancer control group (DMH) had significantly lower leukocyte count compared to INT (*p* < 0.05) and DMH+P (*p* < 0.001) after five months of experiment (Figure 4).

## 4. Discussion

The purpose of this research was to investigate the beneficial effects of a novel panel and mixture of probiotic species with respect to their immunomodulatory capacity on macrophages and their potential to be adopted for prophylaxis of colorectal cancer, a pathology that is associated with macrophage subset-specific effector responses. This study resulted in several important findings, namely: (1) probiotic strains used in this study differentially induced macrophage pro-inflammatory cytokines in a subset- and strain-specific manner, (2) phagocytosis of probiotic was both macrophage subset- and bacterial strain-specific, (3) probiotic mix abrogates DMH-induced shortening of the colon and exhibits a beneficial effect on leukocyte count and colon tumour development, and (4) probiotic mix induces secretion of pro-inflammatory IL-18 by tumour tissue.

In agreement with similar studies, the probiotic bacteria tested in this study induced secretion of pro-inflammatory, hence potentially anti-tumour cytokines (TNF-α, IL-1β, IL-18, IL-23) in a strain-specific and cell-subset specific manner [14,37,38]. M1-like macrophages generally secreted higher levels of TNF-α compared to M2-like (anti-inflammatory) macrophages. In the case of M1s, strains of genus LP were 10 to 20-fold less potent than strains of LS, whereas in M2s, the LP strains C28 and MS18 induced similar levels to the LS strains, indicative of a more pro-inflammatory effect of these bacteria on this subset of macrophage. LP VD23 was shown to have the least potent effect on secretion of TNF-α in M2s. TNF-α, originally described for its anti-tumour properties [39], is a multifunctional cytokine, which, at high doses, can induce tumour cell apoptosis whereas it accelerates tumour progression via tumour invasion and metastasis with chronic low doses [40,41,42,43]. It was also found, that TNF-α concentration increases with advanced stage of CRC [42]. The same trend was exhibited for IL-1β production where LS strains induced 2.5 to 16-fold higher levels of this cytokine compared to LP strains in M1-like cells. LP VD23 showed poor ability to induce secretion of this cytokine in both macrophage subsets tested, however it must be noted that the fold induction was much higher in M2 than M1 macrophages. IL-1β can be considered as both pro- and anti-tumoral; that is, it promotes anti-tumour responses via its pro-inflammatory nature and its ability to promote Th_17_ differentiation and neutrophil activation and it is also able to drive pro-tumour responses via tumour invasiveness and immunosuppression [44,45]. Higher IL-1β levels can induce myeloid-derived suppressor cells (MDSC), which exhibit phenotypic similarity to anti-inflammatory M2 macrophages capable of suppressing anti-tumour immunity, and resulting in tumour growth. MDSCs and M2 macrophages are present at appreciable levels in many different types of cancer including CRC [46]. This would suggest that the probiotic strain-induced cytokine profile can effectively manipulate macrophage subset plasticity and the switching from pro-inflammatory to pro-tumour effector phenotypes. Similar results were observed for IL-18, an IL-1β family cytokine that promotes protective host immunity mediated by CD8^+^ cytotoxic T cells (T_c_), NK cells [47,48] and Th_1_-driven macrophage activation. NK cells exhibit an anti-tumour responses, therefore an increase in IL-18 secretion may be relevant to the anti-tumour activity of specific lactobacilli strains in rat colorectal cancer models [49,50]. In addition, IL-18 is crucial for intestinal epithelial cells’ (IECs) homeostasis and mucosal repair, inducing IEC proliferation/differentiation, goblet cell mucus production, the expression of tight junction proteins and secretion of anti-bacterial peptides [51,52] essential in preventing CRC development [53]. LS species potently induced this cytokine in both cell subsets, whereas LP strains had little or no ability to stimulate IL-18 secretion compared to control. Interestingly, LS MS3 induced 2.5-fold higher production of IL-18 in M2-like cells compared to M1-like cells, which may be indicative of a subset plasticity switch towards a pro-inflammatory/anti-tumour M1 phenotype. IL-23 is a heterodimeric pro-inflammatory cytokine, which, in ulcerative colitis, activates and induces cytokine production of NK cells, intraepithelial lymphocytes (IEL), innate lymphoid cells (ILCs), and T_h_17 (reviewed in [54]), whereas it blocks activation of regulatory T cells (T_reg_) [55]. This pro-inflammatory nature however, is contradicted by several observations where serum IL-23 levels were increased in CRC patients and positively correlated with VEGF [56] and high primary tumour tissue levels of IL-23 were predictive of increased CRC metastasis [57]. LS strains stimulated both macrophage subtypes to secrete this cytokine, whereas strain MS3 potently induced secretion of this cytokine in M2 cells. LP strains, on the other hand, showed little or no induction of IL-23. 

Differential cytokine production profiles is a key feature of polarised macrophages exhibiting differential effector functionality, such as phagocytic capability [58,59]. Homeostatic mucosal macrophages resemble the M2 subset, and are characterised by high phagocytic activity, scavenger receptors (CD36, CD68, CD206) and low pro-inflammatory cytokine production, whereas during inflammation, they polarise towards an M1-like subset (Phago^lo^, Scavenger Receptor^lo^, Pro-inflammatory Cytokine^hi^) [60]. Macrophage phagocytic capability and the nature/health of target cells being recognised for phagocytosis have a profound effect on the macrophage cytokine profile, and hence the plasticity of the effector phenotype [61,62,63]. Phagocytic activity was higher in M2- than M1-like macrophages immediately after the first hour of co-culture with all tested probiotic strains. M1s also exhibited phagocytic activity, but at delayed onset time points. In M2-like cells, the highest phagocytosis was observed after six hours of co-culture. In general, LS MS3 induced low phagocytic activity in both M1s and M2s, whereas the cytokine profile stimulated by this strain in both macrophage subsets was TNF-α^hi^, IL-1β^hi^, IL-18^hi^, IL-23^hi^. This is indicative of the potential of MS3 to drive towards an M1 phenotype characterised by low phagocytic activity and high pro-inflammatory cytokine expression. *Lactobacillus* MS6 also stimulated cytokine secretion to high levels in both macrophage subsets, but interestingly phagocytosis in M1-like cells was the highest for all tested strains. This might indicate that both pro- and anti-inflammatory properties are inducible by this bacterial strain and that macrophage polarisation may be intermediate between conventional M1 and M2 subsets. MS16 potently induced pro-inflammatory cytokines whilst inducing a low to medium level of phagocytosis, which demonstrates the pro-inflammatory potential in both M1s and M2s. Both LP strains MS18 and C28 had poor capability to induce cytokine release and generated medium phagocytic activity in both macrophage subsets, suggesting the more anti-inflammatory capacity of these strains. Finally, both M1s and M2s displayed a TNF-α^lo^, IL-1β^lo^ IL-18^lo^, IL-23^lo^ profile after stimulation by LP VD23 whereas M2s were Phago^hi^ and M1s Phago^med^. Of all the tested lactobacilli, this would appear to be the most anti-inflammatory strain with potentiation of an M2 phenotype. From these data, the tested probiotic bacteria showed pro-, anti- and mixed pro- and anti-inflammatory properties in vitro. It is however, essential to test production of other cytokines, expression of scavenger receptors, Arg-1 and iNOS to confirm the potential of these strains to polarise towards distinct macrophage subsets. Nevertheless, this study has demonstrated an association between probiotic strain LS MS3 and M1 macrophage phenotyping, whereas LP VD23 showed an association with M2 macrophage phenotype. Other strains showed an intermediate polarising capacity between these two distinct macrophage subsets. Consequently, these probiotic bacterial strains may differentially drive functional plasticity between M1 and M2 macrophage subsets, effectively reprogramming macrophage effector functions towards pro-inflammatory/anti-tumour responses or anti-inflammatory/immune suppressive responses.

As a consequence of the in vitro data, which demonstrated a range of probiotic-driven effects such as pro-, anti- and both pro- and anti-inflammatory, and taking into account the differential immune responses exhibited at different stages of tumour progression, the in vivo evaluation of anti-cancer effects of probiotics on a DMH model of colon carcinogenesis utilised a mixture of the six probiotic strains. Studies in experimental CRC models suggest that tumour infiltrating immune cells and their inflammatory cytokines either directly or indirectly stimulate the uncontrolled growth of cancer cells, from neoplastic transformation to tumour expansion and metastasis. The onset and the progress of CRC could be potentially modified using a panel of lactobacilli with different macrophage reprogramming capability. During carcinogenesis, the tested lactobacilli mix, especially the anti-inflammatory M2-programming VD23 strain, ameliorates the inflammatory conditions (in the early stages) and/or the pro-inflammatory M1-programming MS3 strain can boost an anti-tumour immune response with the down-stream effect of eliminating dysplastic and cancerous cells. With respect to long-term study of CRC, where cancer arises from chronic inflammation and leads to an immunosuppressive state with tumour presence, a mixture of probiotic bacteria with both anti- and pro-inflammatory (M2- and M1-programming) features was used, and this may represent a realistic approach to harnessing probiotic strains in the modulation of CRC.

While body weight gain over the experimental period did not differ, there was a significant difference in daily food intake between all experimental groups. Despite the increased food intake of the DMH group compared to the DMH+P group, the rats’ ability to convert food into body mass (expressed by FER) was not significantly affected. The probiotic-fed group was shown to have the highest FER, therefore it can be suggested that probiotic treatment can improve absorption and digestion of food [64]. In general, the improved health status of probiotic-fed cancer animals was obvious. In addition, probiotic treatment in the DMH+P group significantly increased colon length, effectively rescuing the reduced colon length observed in the DMH group. Similar results were found by [65], who reported that probiotic supplementation restored colon length.

Although the incidence and multiplicity of tumours were not significantly influenced by probiotic supplementation, a trend in decreasing tumour volume and numbers in the DMH+P group compared with cancer control was evident. The total number of tumours was 33% less for the DMH+P group, suggesting a positive effect of probiotic treatment. Cytokine profiling in tumour tissue is a good indicator of tumour immunity, tumour development and prognosis [66]. Probiotic treatment had no significant effect on tumour secretion of the pro-inflammatory cytokines, TNF-α and IL-1β, whereas IL-18 was significantly increased in tumour tissue of DMH+P animals. TNF-α and IL-1β exhibit both pro- and anti-tumour activity in CRC, dependent on stage of progression; IL-18 however, is considered an anti-tumorigenic cytokine, that not only affects Th_1_-cell-mediated immunity but also stimulates anti-tumour activity of NK cells, thus, a probiotic-mediated augmentation of IL-18 suggests it had a protective effect in this in vivo system. Better tumour surveillance of probiotic-fed DMH rats can be also linked to this probiotic potential to induce IL-18 secretion, as IL-18 is critically involved in protection against colorectal tumorigenesis [67]. Additionally, IL-18 also induces the expression of CD80, CD86, HLA-DR and HLA-DQ on NK cells derived from cancer patients, suggesting that IL-18 conferred NK cells on an APC-like phenotype, which indicates increased recognition and responsiveness to tumour antigens [68]. As with TNF-α and IL-1β, IL-18 may also exhibit both pro- and anti-tumour activity in CRC, depending on the stage of progression and immune effector cells responsive to IL-18. It was found, that in models of IBD, secreted IL-18 potentiates IL-18 responsiveness via augmentation of *lamina propria* CD4^+^ T cell IL-18 receptor expression and that IL-18 signalling restricts intestinal inflammation by limiting the T_h_17 differentiation and promoting T_reg_ effector responses [69]. 

Leukocyte count slightly decreases with age in rodents and humans [70,71]. The same trend was also observed in the present study with a slight difference between experimental groups. Interestingly, there was a significant decline in leukocyte count after five months of experiment in the DMH group (*p* < 0.05), while leukocyte count in the DMH+P group did not change compared to the INT group. More profound changes in peripheral leukocyte count (especially a decrease in monocytes and lymphocytes count) can be seen in patients where disease has been systematically spread [72], therefore these findings confirm the hypothesis that probiotics can increase quality of life or delay the onset of the disease [73,74]. 

## 5. Conclusions 

Currently there are many studies that support the notion that the microbiome shapes the immune system, effectively modulating tumour development. Moreover, redressing the compositional and metabolic activity dysbiosis of the gut microbiota with probiotics might reduce the risk of carcinogenesis and cancer development. These data confirmed previous findings that each probiotic strain influences innate immune systems in a specific manner. It is crucial to carefully assess the features of each probiotic strain and although probiotic bacteria share some similar properties, it is desirable to avoid generalisations. In this way it will be possible to select the most suitable candidates for therapeutic use based on clinical signs of disease but also personalise them to specific problems in each patient. Strategies for reshaping macrophage polarisation that assist in enhancing the anti-inflammatory activity of M2 macrophages in the early stages of CRC and the tumoricidal activity of M1 in the immunosuppressive later stages, could be a promising therapeutic modality worthy of future consideration in CRC management. Selective probiotic strains can be used prophylactically by inducing M2-driven responses in the early stages of CRC, or by inducing anti-tumour M1-driven responses at the tumour stage of CRC. For future studies, it is vital to test probiotics for their activity in specific stages of the disease. It is clear, that probiotics themselves will not be able to cure cancer, but they can be used to prevent and/or help to treat CRC by acting on the immune system and increasing the efficacy of treatments. 

## Figures and Tables

**Table 1:** The name, accession number, code and origin of lactic acid bacteria (LAB) strains tested in the present study. The code refers to strains name used in the study.

Name.	Accession Number	Code	Origin
*Lactobacillus plantarum* VD23	NCIMB 41608	VD23	isolated from the faeces of pigs [29]
*Lactobacillus plantarum* C28	NCIMB 41605	C28	isolated from the faeces of pigs [29]
*Lactobacillus plantarum* MS18	NCIMB 41607	MS18	isolated from a healthy chicken small intestine [30]
*Lactobacillus salivarius* MS3	NCIMB 41606	MS3	isolated from a healthy chicken small intestine [30]
*Lactobacillus salivarius* MS6	NCIMB 41609	MS6	isolated from a healthy chicken small intestine [30]
*Lactobacillus salivarius* MS16	NCIMB 41610	MS16	isolated from a healthy chicken small intestine [30]

**Table 2:** Probiotic treatment of colorectal cancer (CRC) partially rescued DMH-induced effects on food efficiency ratio (FER) and gut length.

Groups	Body-Weight Gain (g)	Food Intake (g/rat/day)	FER (%) ^1^	Gut Length (cm)
DMH	378.6 ± 13.7	22.5 ± 0.08 ^A^	7.67 ± 0.23 ^a^	18.58 ± 1.04 ^a^
DMH+P	350.3 ± 11.1	21.5 ± 0.08 ^B^	8.09 ± 0.25 ^ab^	21.3 ± 0.6 ^b^
P	400.5 ± 16	23.3 ± 0.07 ^C^	8.68 ± 0.31 ^b^	21.5 ± 0.52 ^b^
INT	404.6 ± 17.7	24.2 ± 0.09 ^D^	7.78 ± 0.21 ^a^	21.2 ± 0.52 ^b^

All values are expressed as mean ± SEM. Means with the same superscript are not significantly different; different superscript letters are significant, where *p* < 0.05 (small letters) and *p* < 0.001 (when superscript in capitals). ^1^ FER (food efficiency ratio) (%) = (body-weight gain/food intake) ×100.

**Table 3:** Effect of LAB mixture on incidence, multiplicity, number and volume of tumours in the colon of experimental animals.

Groups	Tumour Incidence	Tumour Multiplicity ^1^	Total Number of Tumours	Numbers of Tumours in Proximal Colon	Numbers of Tumours in Distal Colon	Average Tumour Volume (cm^3^) ^1^
DMH	4/8 (50%)	2.25 ± 1.25	9	7	2	2.42 ± 0.56
DMH+P	4/8 (50%)	1.5 ± 0.29	6	5	1	1.66 ± 0.64
P	0/8 (0%)	0	0	0	0	0
INT	0/8 (0%)	0	0	0	0	0

^1^ Values for tumour multiplicity and average tumour volume are expressed as mean ± SEM.
